# Method of preparation, visualization and ultrastructural analysis of a formulation of probiotic *Bacillus subtilis* KATMIRA1933 produced by solid-phase fermentation

**DOI:** 10.1016/j.mex.2019.10.030

**Published:** 2019-10-31

**Authors:** G.M. Fedorenko, A.G. Fedorenko, V.A. Chistyakov, E.V. Prazdnova, A.V. Usatov, M.L. Chikindas, M.S. Mazanko, R. Weeks

**Affiliations:** a Southern Federal University, Russia; b Health Promoting Naturals, School of Environmental and Biological Sciences, Rutgers University, United States

**Keywords:** Method of preparation, visualization and ultrastructural analysis of a formulation of probiotic *Bacillus subtilis* KATMIRA1933 produced by solid-phase fermentation, Electron microscopy, Solid-phase fermentation, Bacillus probiotic

## Abstract

Probiotic preparations are used in medical treatment and in agricultural practice. They modulate numerous activities in eukaryotic hosts, such as: inhibition of pathogenic microbiota; stimulation of immunological responses; and production of antioxidants, anti-mutagens, and DNA protectors. Also, probiotic bacteria are used as a preventive measure to prevent bacterial diseases of the gastrointestinal tract. Solid-phase fermentation is reported as being used in the production of probiotic formulations where a solid substratum, such as soy and oil meal, is utilized for the growth of beneficial microorganisms. However, there are insufficient reports in the literature related to methodological approaches enabling evaluation of the final products of solid-phase fermentation.

We suggest a novel method enabling evaluation of probiotic solid-state fermentation dry powders and observation of their morphology, ultrastructure, and elucidation of the quantitative distribution of probiotic microorganisms in solid substrates using electron microscopy.

• The method is intended for ultrastructure microphotography of dry substances - for example, ultrastructure of solid-phase fermentation products.
• The method allows preserving the ultrastructure of substrates that are damaged when soaking.
• The method does not require additional equipment and reagents and can be used in all laboratories using electron microscopy.

## Graphical abstract

**Figure:**
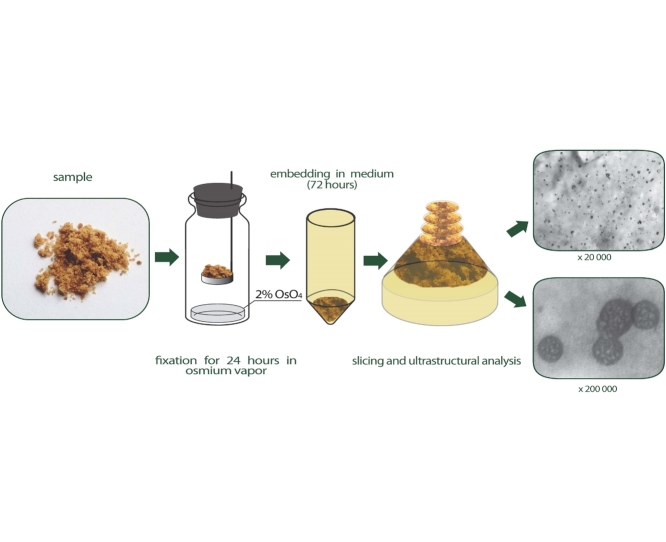

**Specification Table**

**Table:** 

Subject Area:	Agricultural and Biological Sciences
More specific subject area:	Describe narrower subject area
Method name:	Method of preparation, visualization and ultrastructural analysis of a formulation of probiotic *Bacillus subtilis* KATMIRA1933 produced by solid-phase fermentation
Name and reference of original method:	Scanning electron microscopy J.T. SanAgustin, J.A. Follit, G. Hendricks, G.J. Pazour, Chapter 4 - Scanning Electron Microscopy to Examine Cells and Organs, in: S.M. King, G.J. Pazour (Eds.), Methods in Cell Biology, Academic Press, 2009: pp. 81–87. doi: https://doi.org/10.1016/S0091-679X(08)91004-9.
Resource availability:	2% osmium tetroxide (SPI Supplies CAS # 20816-12-0) 100% acetone Epon812 embedding medium (SPI Supplies 02660-AB) Methylene blue solution (SPI Supplies CAS # 7220-79-3 C.I. 52015) Glass slides Copper mesh for TEM (SPI Supplies # 2010C-XA) Glass vial with lid tightly · Ultramicrotome (Leica EM UC6 or similar) Transmission electron microscope (Tecnai G2 Spirit BioTwin Microscope or similar) Thermo cabinet (temperature up to +62 C0)

## Method details

### Background

The traditional methods of sample preparation for transmission electron microscopy (TEM) utilize fixing solutions made with water as a solvent [[1], [2], [3], [4]]. However, in the case of solid-phase fermentation the final product is a dry powder. Therefore, should water be used as a solvent, the sample preparation process may cause the sample’s hydration, leading to the damage of the sample’s native structure. In turn, this may produce unreliable images not reflecting the actual structure of the dry preparation.

The new method prevents any contact of the sample with the water-containing part of the fixing solution.

### Protocol

#### Preparation of probiotic bacterial culture

Probiotic bacteria *Bacillus subtilis* KATMIRA1933, the fermented milk product isolate [5], was used. One kilogram (dry weight) of the Don-21 variety of soybean (39% protein, purchased from the I.G. Kalinenko All-Russia Research Institute of Crops, Zernograd, Rostov Region, Russia) was washed with tap water and then soaked overnight in two volumes of tap water The following day, the water was drained, and the beans were autoclaved for 15 min at 121 °C. The beans were cooled down to 60 °C, and 10 mL overnight culture of *B. subtilis* KATMIRA1933 (10^8^ CFU/mL) in LB medium were added and mixed thoroughly. The inoculated beans were placed in a layer of 3–5 cm in a sterile tray with a non-hermetic lid and incubated for 24 h at 42 °C.

Then the beans (50% moisture), coated with bacterial biofilm (3 × 10^11^ CFU/g), were ground in a meat grinder (Kenwood 700 MG, Watford, UK). The resulting mass was placed in open trays in a layer of 1–2 cm and incubated for 48–72 hours at 45 °C in a ventilated incubation chamber (VD720, Binder, Germany), until dried (5% moisture). After drying, the solid product granules were milled in a coffee grinder in aseptic conditions (KMM 30, Braun, Kronberg, Germany) [6].

#### Fixation and imbibition in pouring substratum

**Fig. 1:**
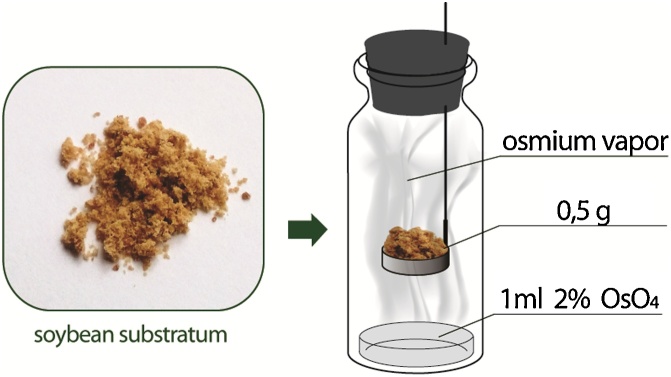
Fixation without any contact with the water fraction of a fixating solvent.

Soy substrate-grown probiotic formulation (SSPF) was placed in a special glass vial with a well-closed bung (Fig. 1). Then 1 mL of a 2% solution of OsO_4_ (SPI Supplies CAS# 20816-12-0) was added. The tray with SSPF was placed above the solution level, and was incubated for 24 h at room temperature. Then the samples were transferred into a pouring mixture Epon 812 [epoxipropyl ether of glycerol, epon] (SPI Supplies 02660-AB) [1]. For proper pouring of the SSPF, it is important to do the procedure stepwise in the epone-acetone mixture, with a gradually increasing concentration of epon. The next step, the polymerization stage, takes place for 24 h at 37 °C, and then continues for 48 h at 62 °C.

Use the following turn-based protocol for the sample fixing and pouring:

1 Add 1 mL of a 2% solution of OsO_4_ in a glass vial. Then place 0.5 g of SSPF on the tray (Fig. 1), close the vial hermetically (osmium vapors are volatile) and leave it at 20 °C for 24 h.
2 Remove the SSPF sample from the vial and place it in the acetone : epon (2:1) mixture (1 ml) for 90 min.
3 Remove the liquid phase, add the acetone: epon mixture (1:1) (1 ml) and incubate for 90 min.
4 Remove the solvent and replace it with acetone: epon (1:2) (1 ml) for 90 min.
5 Discard the solvent and replace it with 100% epon (1 ml) for 60 min.
6 Carefully remove the epon, then place the SSPF sample into a conic capsule for pouring and incubate in a thermostat for 24 h at 37 °C, and then for 48 h at 62 °C.

Microslip preparation for light and electron microscopy:

1 Remove the epon-polymerized SSPF preparation from a capsule using scissors or lancet.
2 Place the SSPF polymerized sample into the ultramicrotome adapter and shape it into a pyramid using the razor blade.
3 Make ‘semi-thin’ microslips (700 nm) using an ultramicrotome (e.g. Leica EM UC6) and place them in a distilled water drop, on the slide.
4 Fix microslips onto the slide with flame (avoid overheating!).
5 Dye microslips with a 1% solution of methylene blue (SPI Supplies CAS #7220-79-3 C.I. 52015) [4]
6 Dry the slide with flame, again, avoid boiling the liquid.
7 Wash microslips carefully with distilled water. The microslips are now ready for light microscopy.
8 Identify the part of the sample selected for microscopic observation; if necessary, correct the pyramid, and make ultrathin microslips (70–90 nm).
9 Place the microslips on a copper lattice for TEM (SPI Supplies #2010C-XA).
10 The microslips are now ready for electron microscopy. Images presented in this paper were obtained using a Tecnai G2 Spirit BioTwin microscope (FEI Company, Hillsboro, OR).

#### Method validation

**Fig. 2:**
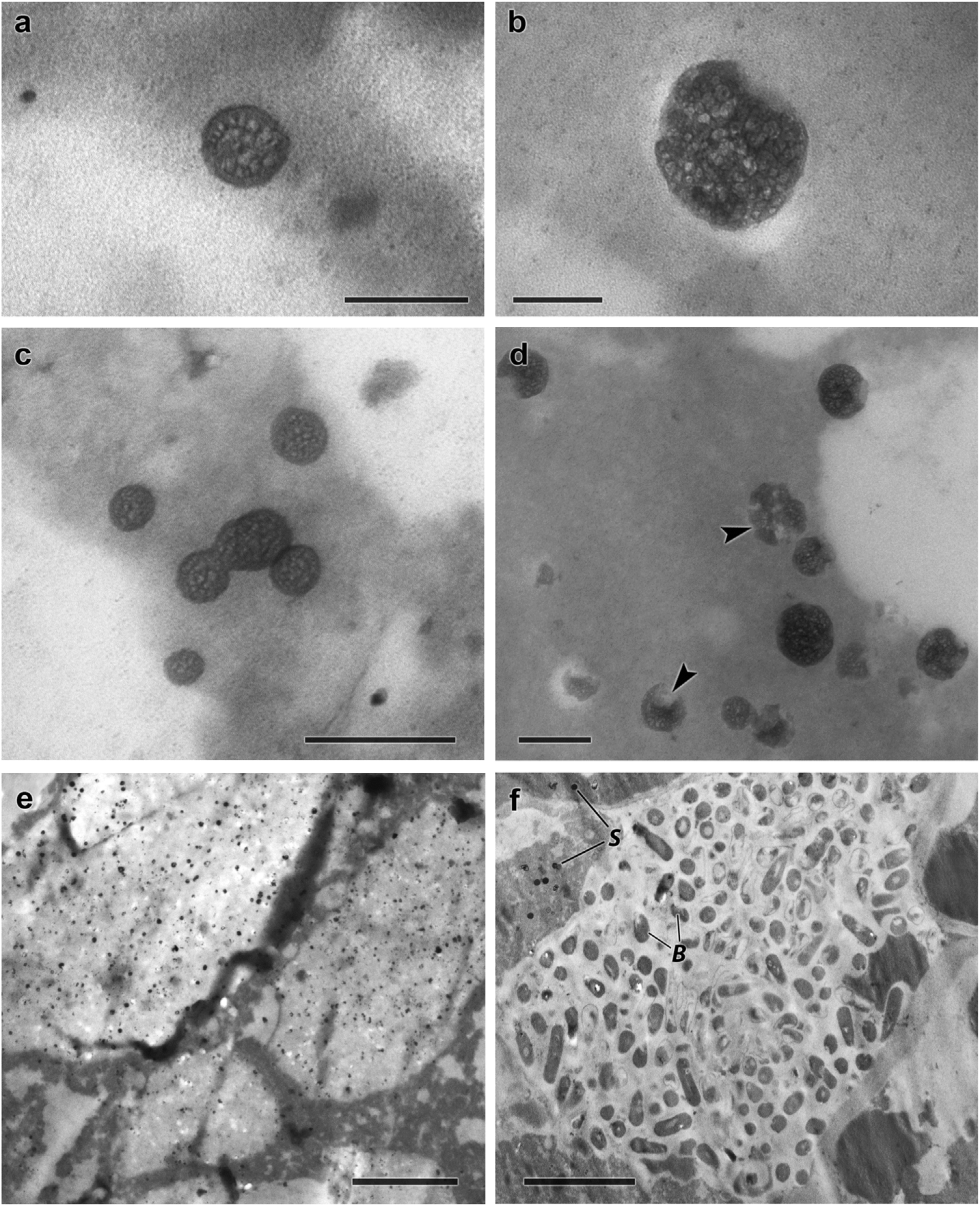
Spores of bacteria *B. subtilis* KATMIRA1933. **a, c, e** – fixation in vapour of OsO_4_. **b, d, f** – double glutaraldehyde-osmium fixation, S – spores, B – bacterial cells, arrows are damaged spores. The scale segment: **a, b –** 100 nm, **c, d –** 200 nm, **e –** 2 μm, **f –** 5 μm.

The method presented here is a reliable approach for producing electron microphotographs of the final products of solid-phase fermentation, which are dry powders. The size of *B. subtilis* KATMIRA1933 spores is less than 100 nm, which makes TEM one of a few methods allowing visualization of dry samples. To verify this method, we compared our electron microphotographs with those obtained using traditional fixing in glutaraldehyde and further treatment with OsO_4_ (Fig. 2). Although ‘double fixing’ produces a somewhat more reliable sample stabilization in the majority of the cases [1], it is evident from our comparison that the decrease in the fixing agent’s quantity had no negative impact on the ultrastructure’s quality, contrast, and electron density. At the same time, our method allowed for better preservation of the samples ultrastructure. Interestingly, the size of the spores in the samples prepared according to the traditional ‘double fixing’ procedure are 2–3 times bigger than their size in the samples prepared with OsO_4_ vapor fixation. The increased size of the spores can probably be explained by their hydration. The spore size becomes bigger than the microslip’s gage, which may cause damage during the microslip preparation. Ultimately, this may result in electron microphotographs with unreliable images of damaged spores and an interrupted microstructure in the sample. Our method of solid sample preparation avoids these undesirable effects.

#### Additional information

The World Health Organization defines probiotics as live microorganisms capable of delivering scientifically measurable positive effects to eukaryotic organisms, when administered in adequate quantities [7]. Probiotic preparations are used in medical treatments, over-the-counter formulations and in agricultural practice. Probiotics have a positive influence on animal and human organisms: they inhibit pathogenic microbiota [8], stimulate immunological reactions [9], and help to consolidate the cytoskeleton of intestinal epithelium cells, reducing the permeability of the intestinal mucosa [10,11]. Probiotics are reported as producers of antioxidants, anti-mutagens, and DNA protectors [[12], [13], [14]]. These justify the attention to probiotic formulations and their use as medicinal formulations and in dairy products [[13], [14], [15], [16], [17]].

Unlike non-sporeforming probiotics (e.g. strains of lactobacilli and bifidobacteria), sporeformers have a better survival rate when passing through the highly acidic environment of stomach and small intestine, and are more stable during product manufacturing and storage [12,18].

The science and technology of solid phase production of probiotics is presently on the radar of many investigators from industry and academia. Unlike traditional liquid fermentation, the substratum for bacterial growth is solid, such as soybeans. Bacterial cells grow on these substratums in the form of biofilms. This method differs from other methods by its simplicity, environmental-friendliness, and economic efficiency [12,[19], [20], [21]].

Our studies demonstrated that sporeforming probiotic organisms, obtained through solid phase fermentation, have antimutagenetic and antioxidative effects, as well as increase chicken mass and egg-laying ability [12,22,23].

## Declaration of Competing Interest

The authors declare that they have no known competing financial interests or personal relationships that could have appeared to influence the work reported in this paper.
